# Three-Year Outcomes Following Permissive Cardiotoxicity in Patients on Trastuzumab

**DOI:** 10.1093/oncolo/oyad086

**Published:** 2023-04-24

**Authors:** Shijie Zhou, Filipe Cirne, Justin Chow, Arman Zereshkian, Louise Bordeleau, Sukhbinder Dhesy-Thind, Peter M Ellis, Som D Mukherjee, Nazanin Aghel, Darryl P Leong

**Affiliations:** Department of Medicine, McMaster University, Hamilton, Ontario, Canada; Department of Medicine, McMaster University, Hamilton, Ontario, Canada; Department of Medicine, McMaster University, Hamilton, Ontario, Canada; Department of Medicine, McMaster University, Hamilton, Ontario, Canada; Department of Oncology, Department of Health Research Methods, Evidence, and Impact, McMaster University, Hamilton, Ontario, Canada; Department of Oncology, McMaster University, Hamilton, Ontario, Canada; Department of Oncology, Department of Health Research Methods, Evidence, and Impact, McMaster University, Hamilton, Ontario, Canada; Department of Oncology, McMaster University, Hamilton, Ontario, Canada; Department of Oncology, McMaster University, Hamilton, Ontario, Canada; Population Health Research Institute and Department of Medicine, McMaster University, Hamilton, Ontario, Canada

**Keywords:** cardiotoxic agents, cardio-oncology, trastuzumab, retrospective studies, permissive cardiotoxicity

## Abstract

**Introduction:**

Cardiotoxicity, manifest by reduced left ventricular ejection fraction (LVEF), is the most common reason for the premature discontinuation of trastuzumab. While permissive cardiotoxicity (where mild cardiotoxicity is accepted to enable ongoing trastuzumab) has been shown feasible, the longer-term outcomes are unknown. We aimed to study the intermediate-term clinical outcomes of patients who underwent permissive cardiotoxicity.

**Materials and Methods:**

We performed a retrospective cohort study of patients referred to the cardio-oncology service at McMaster University from 2016 to 2021 for LV dysfunction following trastuzumab administration.

**Results:**

Fifty-one patients underwent permissive cardiotoxicity. The median (25th-75th percentile) follow-up time from cardiotoxicity onset was 3 years (1.3-4 years). Forty-seven (92%) patients completed trastuzumab; 3 (6%) developed severe LV dysfunction or clinical heart failure (HF) while on trastuzumab and prematurely discontinued therapy. One discontinued trastuzumab by patient choice. At final follow-up after therapy completion, 7 (14%) patients still had mild cardiotoxicity, including 2 who had clinical heart failure and stopped trastuzumab early. Among those with recovered LV function, 50% had normalized LVEF or GLS by 6 and 3 months, respectively, after initial cardiotoxicity. There was no difference in characteristics between those who did or did not recover their LV function.

**Conclusions:**

Among patients exposed to permissive trastuzumab cardiotoxicity for HER2-positive breast cancer, 6% were unable to complete planned trastuzumab due to severe LV dysfunction or clinical HF. Although most patients recover their LV function after trastuzumab discontinuation or completion, 14% still have persistent cardiotoxicity by 3-year follow-up.

## Teaser

This article reports oncological and cardiac outcomes of 51 patients with HER2-positive breast cancer who developed mild cardiac dysfunction after trastuzumab administration, and pursued a strategy of permissive cardiotoxicity.

**Implications for Practice**

In patients with HER2-positive breast cancer who developed cardiotoxicity on trastuzumab, more than 90% were able to continue trastuzumab under a strategy of permissive cardiotoxicity without clinical heart failure or severe LV dysfunction. Importantly, by a median follow-up of 3 years, 14% had evidence of persistent LV dysfunction. A strategy of permissive trastuzumab cardiotoxicity is feasible in most patients without any adverse outcomes in the intermediate term. Future studies are needed to study the long-term safety of such a strategy and factors associated with intolerance of permissive cardiotoxicity.

## Introduction

Breast cancer is the second leading cause of cancer mortality in women. Among breast cancers, 20%-25% overexpress the human epidermal growth factor 2 (HER2) receptor that increases tumor aggressiveness.^1^ HER2-targeted therapies improve pathologic complete remission rate, overall- and disease-free survival in patients with HER2-positive breast cancer.^2,3^

Cardiotoxicity is the most common dose-limiting complication of HER2-targeted therapies, such as trastuzumab. Approximately 1 in 5 patients receiving adjuvant trastuzumab experiences asymptomatic but clinically relevant decline in left ventricular systolic function, and up to 4% of patients suffer moderate to severe heart failure.^4^ Regulatory approvals for trastuzumab require that trastuzumab be withheld for substantial deterioration in left ventricular ejection fraction (LVEF) and permanently discontinued if LVEF fails to normalize during >8 weeks.^5-8^ However, several randomized, controlled clinical trials have shown that a shorter duration of trastuzumab therapy (as compared to a standard 12-month course) led to increased cancer mortality.^9^ Similarly, Sardesai et al showed that patients who had any interruption to trastuzumab (62% due to cardiotoxicity) had worse overall survival (adjusted HR of 4.8) and disease-free survival (adjusted HR of 4.4) after accounting for confounders.^10^ Therefore, there has been increasing interest in a strategy of permissive cardiotoxicity, whereby HER2-targeted therapy is continued as long as LVEF is only mildly impaired, and the patient remains minimally symptomatic.^4,11^ Based on experience in 50 patients followed for approximately one year, the recommendations in the 2022 European Society of Cardiology Cardio-Oncology guidelines are that patients with asymptomatic mild-to-moderate cancer therapy-related cardiac dysfunction should be considered for ongoing HER2-targeted treatment with concurrent cardioprotective therapy and close multi-disciplinary monitoring.^12^ However, in the absence of a large body of evidence and longer-term follow-up, there remains uncertainty about the risks and benefits of implementing this recommendation. Therefore, we undertook a retrospective study of consecutive patients with HER2-positive breast cancer referred to a dedicated cardio-oncology service in a quaternary care institution managed using a strategy of permissive cardiotoxicity. The primary objective of this study was to describe the intermediate-term clinical outcomes and echocardiographic findings in this population. The secondary objective was to characterize patients who developed cardiac dose-limiting toxicity (cDLT) when undertaking this therapeutic approach.

## Methods

### Study Design

We performed a retrospective cohort study of patients referred to the cardio-oncology service, McMaster University, Hamilton, Canada due to left ventricular (LV) dysfunction following trastuzumab administration. The study was approved by the local ethics committee (Hamilton Integrated Research Ethics Board).

Patients were eligible for inclusion if referred between January 2016 and May 2021, with a history of HER2 positive stages I–IV breast cancer treated with trastuzumab, who demonstrated cardiotoxicity on echocardiography or multigated acquisition imaging. We defined cardiotoxicity according to the 2008 Canadian Trastuzumab Working Group recommendations, which were current at the time referrals to our clinic began^7^:

1. LVEF within normal limits (≥54% in women) and an absolute fall in LV ejection fraction of >15% from baseline; or
2. LVEF below lower limit of normal (54%) and an absolute fall of ≥10% from baseline

In 2021, the International Cardio-Oncology Society (ICOS) issued new definitions of cardiotoxicity.^13^ Patients were also eligible if they met any of these criteria:

1. Mild dysfunction: LVEF ≥50% and new relative decline in GLS by >15% from baseline.
2. Moderate dysfunction: new LVEF drop ≥10% to 40%-49%, or new LVEF reduction <10% to 40%-49% and new relative decline in GLS by >15% from baseline.
3. Severe dysfunction: new LVEF reduction to <40%.

We included patients who agreed to pursue a strategy of permissive trastuzumab cardiotoxicity, defined as the administration of trastuzumab despite cardiotoxicity as previously defined. All patients were prescribed pharmacotherapies such as angiotensin converting enzyme inhibitor (ACEI), angiotensin receptor blocker (ARB), and beta-blocker (BB) as tolerated. Patients who participated in the SCHOLAR study, a prospective clinical trial of permissive cardiotoxicity conducted at McMaster University, were also included in the current study.^14^

For each included patient, we extracted data on demographics, cancer hormone receptor status, breast cancer stage, anthracycline exposure, left-sided radiation, other HER2-targeted therapies, cardiac risk factors (hypertension, dyslipidemia, diabetes, smoking, coronary artery disease, family history of cardiovascular disease, and alcohol consumption), prior history of cardiomyopathy with or without recovery, physical exam findings, and dosing of cardioprotective medications such as an angiotensin-converting enzyme inhibitor (ACEI), angiotensin receptor blocker (ARB), or a beta-blocker (BB).

Patients treated with permissive trastuzumab cardiotoxicity, who were unable to tolerate further trastuzumab according to clinical judgement, owing to either heart failure symptoms or worsening LV systolic dysfunction (LVEF <35%), were deemed to have experienced cardiac ­dose-limiting toxicity (cDLT).^14^

### Study Outcomes

The primary cardiovascular outcome of interest was LV function as measured in clinically indicated imaging by LVEF and global longitudinal strain (GLS). Measurements by transthoracic echocardiography were preferred over multigated acquisition imaging (MUGA) when available at the time of referral by treating oncologists, after which patients were followed with serial echocardiograms. All imaging was performed as clinically indicated. When available, LVEF by 3-dimensional imaging was preferred; otherwise, Simpson’s bi-plane LVEF was used. Measurements from up to 8 time points were included:

- Before trastuzumab exposure (baseline).
- The onset of cardiotoxicity resulting in a referral to the cardio-oncology service (index event—point 2).
- Up to 3 time points (every 3 months if stable) after the initiation of cardioprotective medications to accompany ongoing trastuzumab (points 3-5).
- Up to 3 time points after trastuzumab was completed (points 6-8).

Secondary cardiovascular outcomes included:

- Moderate or severe heart failure defined as NYHA Class III or greater.
- Hospitalization related to heart failure.
- Cardiovascular death.

The primary oncological outcome of interest was completion of a full course of trastuzumab therapy, considered as ≥17 doses, equivalent to one-year of treatment per standard adjuvant protocol for non-metastatic HER2 positive breast cancer.^15^ The total dose, as opposed to total time, was used to mark treatment completion due to delay in therapy administration in some patients, resulting in longer than one-year of therapy. In addition, this threshold was considered as complete therapy (even though longer treatment durations may be preferred in patients with metastatic disease) to provide a standardized index of treatment completion (i.e., with a common denominator for both non-metastatic and metastatic disease).

Secondary oncological outcomes included:

- disease progression as defined by the treating oncologist, including local or regional disease progression, or new metastatic disease.
- death from breast cancer or its complications.

### Statistical Analysis

Continuous variables are summarized as means with SD or median (25th-75th percentiles). Categorical variables are summarized as counts and proportions. Categorical variables were compared using Fisher’s exact test. Continuous variables were compared using Student’s t test . Changes in LVEF and strain were evaluated using linear mixed effects models in which patient identity was included as a random effect to account for ­within-individual correlation of measures of left ventricular function. A *P*-value of ≤ .05 indicated statistical significance. All statistical analyses were performed using the STATA software application (version 15.1, College Station, Texas).

## Results

We included 51 consecutive patients referred to the ­cardio-oncology service who had received HER2-targeted therapy, exhibited evidence of LV systolic dysfunction, and agreed to pursue a strategy of permissive cardiotoxicity. Patients were followed for a median (25th-75th percentile) 3 (1.3-4) years from developing cardiotoxicity, during which they had a median (25th-75th percentile) 6 (4-8) cardio-oncology clinic visits.

Patient characteristics are displayed in Table 1. Cancer stage according to AJCC staging was I in 11 (22%), II in 19 (37%), III in 13 (25%), and IV in 7 (14%), unavailable in 1 (2%). Twenty-one (50%) received left-sided radiation. Forty-seven (92%) patients received anthracycline-based chemotherapy of similar cumulative dose. All but 2 received anthracycline sequentially with HER2-targeted therapy: 1 patient had anthracycline exposure for a childhood non-breast cancer 18 years prior to her breast cancer diagnosis, while another patient received epirubicin (doxorubicin equitoxic dose for cardiotoxicity calculated) for a previous breast cancer over 10 years ago.^16,17^ The median (25th-75th percentiles) time from the last dose of anthracycline to the start of current trastuzumab therapy was 35 days (28 days-56 days) for patients with non-metastatic cancer, and 5.7 years (38 days-12 years) for patients with metastatic disease. Nine (18%) patients also received other HER2-targeted therapies, including all 7 patients with metastatic disease. Nine (18%) patients received pertuzumab concurrently with trastuzumab. Four (8%) patients received trastuzumab emtansine (T-DM1) for metastatic disease after trastuzumab was completed.

**Table 1.** Baseline patient characteristics.

	Overall *N* = 51 (100%)	Cardiac dose-limiting toxicity *N* = 3 (6%)	Completed HER2-targeted therapy without cDLT *N* = 47 (92%)	*P*-value
Age	56 ± 11	55 ± 18	56 ± 10	0.84
Left-sided radiation	21 (50)	1 (2)	20 (39)	0.78
Cancer stage at diagnosis				
				
III	13 (25)	2 (67)	11 (22)	
IV	7 (14)	0	7 (14)	
				
Unavailable	1 (2)	0	1 (2)	
Received anthracycline-based therapies	47 (92)	3(6)	43(84)	0.61
Cumulative doxorubicin-equivalent dose, mg/m^2^	244 ± 47	343 ± 145	237 ± 22	<0.001
BMI, kg/m^2^	29 ± 7	27.0 ± 0.2	29.2 ± 7.4	0.54
Systolic BP, mmHg	123 ± 13	126 ± 9	123 ± 13	0.67
Diastolic BP, mmHg	75 ± 7	79 ± 2	75 ± 7	0.34
Coronary artery disease	4(8)	0(0)	4(8)	0.61
Alcohol >10 drinks/week	3 (6)	1 (2)	2 (4)	0.04
Current smoking	3 (6)	0 (0)	3 (6)	0.66
Dyslipidemia	9 (18)	1 (2)	8 (16)	0.49
Hypertension	13 (25)	2 (4)	11 (22)	0.10
Diabetes mellitus	4 (8)	1 (2)	3 (6)	0.10
Any cardiovascular risk factor^1^	24 (47)	3 (6)	21 (41)	0.47
Time from initiation of trastuzumab to onset of cardiotoxicity (days)	155 ± 133	140 ± 55	157 ± 138	0.84
GLS pre-HER2 targeted therapy, %	−18 ± 3	−20 ± 3	−18 ± 3	0.34
LVEF pre-HER2 targeted therapy, %	58 ± 5	60 ± 6	58 ± 5	0.41
LVEF at cardio-oncology referral	48 ± 4	45 ± 5	48 ± 4	0.13
LVESVi pre-HER2 targeted therapy	20 ± 6	20 ± 7	20 ± 5	0.98
TAPSE, cm	2 ± 0.3	2 ± 0.3	2 ± 0.4	0.61
≥Moderate valve disease pre-HER2 targeted therapy^2^	1 (2)	1 (2)4	0 (0)	<0.001

Patient baseline characteristics stratified by the occurrence of cardiac dose-limiting toxicity (cDLT). The patient who elected not complete HER2-targeted therapy (but did not have cDLT) is not represented in the group-wise comparison.

^1^Cardiovascular risk factors include the presence of hypertension, coronary artery disease, diabetes mellitus, smoking, alcohol use.

^2^The patient had pre-existing mitral regurgitation.

Abbreviations: LVESVi, left ventricular end systolic volume, indexed by body-surface area (m^2^); TAPSE, tricuspid annular plane systolic excursion.

Four (8%) individuals had a history of cardiomyopathy: 1 of ischemic etiology (pre-trastuzumab LVEF of 56.4%), 2 from previous anthracycline exposure (pre-trastuzumab LVEF of 60% and 42.5%), and 1 subclinical LV dysfunction (depressed GLS of −14.9% and normal EF) on routine pre-chemotherapy echocardiogram.

### Cardiovascular Outcomes During Permissive Cardiotoxicity

The median (25th-75th percentile) follow-up time from the date of index assessment in the cardio-oncology service to the date of last contact was 3 years (1.3-4 years). Three (6%) patients discontinued HER2-targeted therapy prematurely owing to cDLT and 47 (92%) patients completed HER2-targeted therapy without cDLT. One (2%) individual discontinued HER2-targeted therapy due to patient preference, despite clinically tolerating permissive toxicity; she was not included in any comparative analysis. Supplementary Fig. S1 illustrates the study population by cardiovascular outcomes.

Of the 47 patients without cDLT, 40 had non-metastatic disease and finished all 17 cycles of trastuzumab; 7 had metastatic disease with a median trastuzumab cycles of 24. Among the 3 patients who developed cDLT, none had metastatic disease and the median (25th-75th percentile) number of trastuzumab cycles administered was 6 (4-8).

None of the 10 patients receiving pertuzumab or T-DM1 had discontinuation of HER2-targeted therapy due to cardiotoxicity.

Of the 3 patients who developed cDLT, 1 patient had ­cardiac-related hospitalization (acute pulmonary edema) while the other 2 stopped trastuzumab due to progressively deteriorating LV function; none received any other anti-HER2 therapy. The worst NYHA class during office visits was I, II, and III in each patient. Characteristics of patients who developed cDLT are outlined in Table 2.

**Table 2.** Characteristics of patients who experienced cDLT.

ID	Age	Number of trastuzumab cycle	Baseline LVEF (%)	Nadir LVEF (%)	Relative GLS drop (%)	Hospitali- zation	Worst NYHA	Metastasis/ progression	Cumulative anthracycline dose (mg/m^2^)	Reason for permanent trastuzumab discontinuation
43	65	9	67.3	46.7	24.3	Yes	II	No	486^*^	Symptomatic severe mitral regurgitation
23	65	9	58.2	25.9	62	Yes	III	No	266	Continuous worsening of LV function with multi-valvular abnormalities
29	35	12	55	28	47	No	I	No	253	Continuous worsening of LV function

*Note*: Oncological outcomes and cardiac characteristics of patients who experience cardiac dose-limiting toxicity (cDLT).

^*^Patient received high-dose epirubicin as part of the CEF protocol which is no longer in use.^17^ Epirubicin dose was converted to cardiotoxicity-equivalent doxorubicin dose.^16^

In comparison, among those who did not have cDLT, 4 (8%) developed NYHA class II symptoms; 2 (4%) developed NYHA class III symptoms that improved to NYHA classes I-II with either a temporary suspension of trastuzumab or concurrent diuretics; neither required permanent discontinuation of trastuzumab. No patient developed NYHA class IV symptoms.

During follow-up, 49 (96%) patients were on either an ACEI or ARB; 43 (84%) patients were on a BB; 43 (84%) were on both agents. Two (4%) patients took neither class of medication.

### Cardiovascular Outcomes After Permissive Cardiotoxicity

At final follow-up, 14% (7) of patients still exhibited cardiotoxicity according to ICOS criteria. None had metastatic disease. There was no significant difference in most baseline characteristics between those who recovered LV function and those with persistent cardiotoxicity (Supplementary Table S1). While mean anthracycline dose and moderate to severe valvular dysfunction were higher in those with persistent LV dysfunction, this was mostly driven by a single individual.

The clinical characteristics of those with persistent LV dysfunction are displayed in Table 3. None had pre-existing cardiomyopathy. Patients who experienced cDLT while on anti-HER2 therapy more frequently demonstrated ongoing cardiotoxicity at the end of follow-up as compared with those who did not experience cDLT (67% vs. 11%, *P* = .01). Four individuals had no heart failure symptoms during follow-up. Three were symptomatic during and after trastuzumab therapies, including 2 with cDLT.

**Table 3.** LV function in patients who had persistent cardiotoxicity after trastuzumab completion or discontinuation.

ID	Age	Baseline GLS	Nadir GLS	GLS at final follow-up	Baseline LVEF	Nadir LVEF	LVEF at final follow-up	Worst NYHA	Follow-up (days)	Pre-existing cardio-myopathy	Discontinued trastuzumab prematurely due to cDLT
16	62	−21.8	−15	−16.9	58	50	51	I	155	No	No
23	65	−17.8	−6.8	−12.9	58	26	58	III	1416	No	Yes
28	68	−20.2	−14.1	−14.9	58	50	53	I	602	No	No
37	41	N/A	−12.4	−16.6	58	45	47	II	759	No	No
38	64	−21.8	11.5	−16.7	61	38	53	I	974	No	No
39	69	N/A	−14.1	−14.2	60	45	49	I	155	No	No
43	65	−22.6	−16.9	−16.9	67	45	56	II	256	No	Yes

### LV Systolic Function

**Figure 1.**
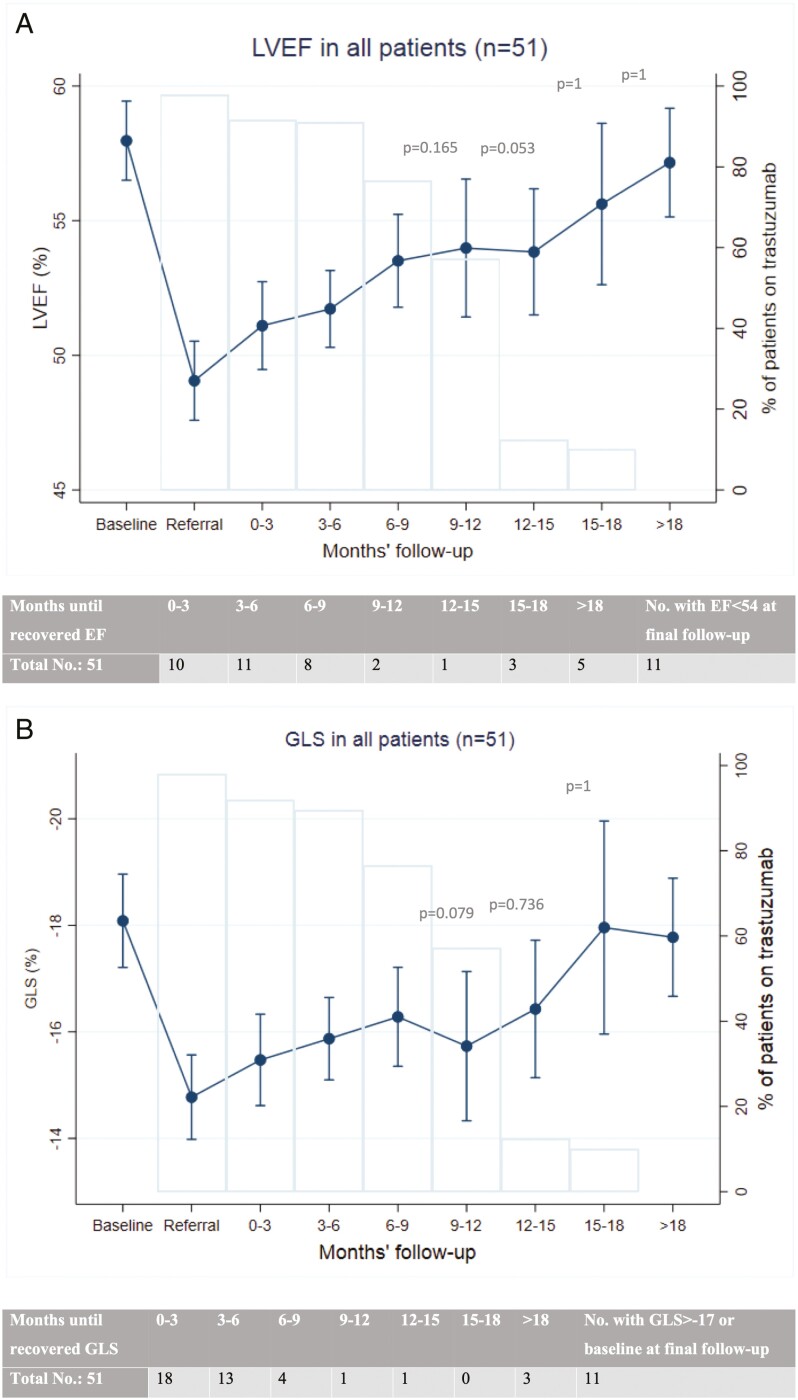
Left ventricular ejection fraction (LVEF) and global longitudinal strain (GLS) in all patients. The graphs show LVEF (**a**) and GLS (**b**, scale reversed for ease of interpretation) in all patients (*n* = 51) in the study. Bars represent percent of patients on trastuzumab at each time point. Table accompanying each graph shows the number of patients who demonstrated normalized LVEF (≥54%) or normalized GLS (≤-17% or to baseline level) at each time point. *P*-values represent the comparison of LVEF or GLS with baseline values at selected time points.

**Figure 2.**
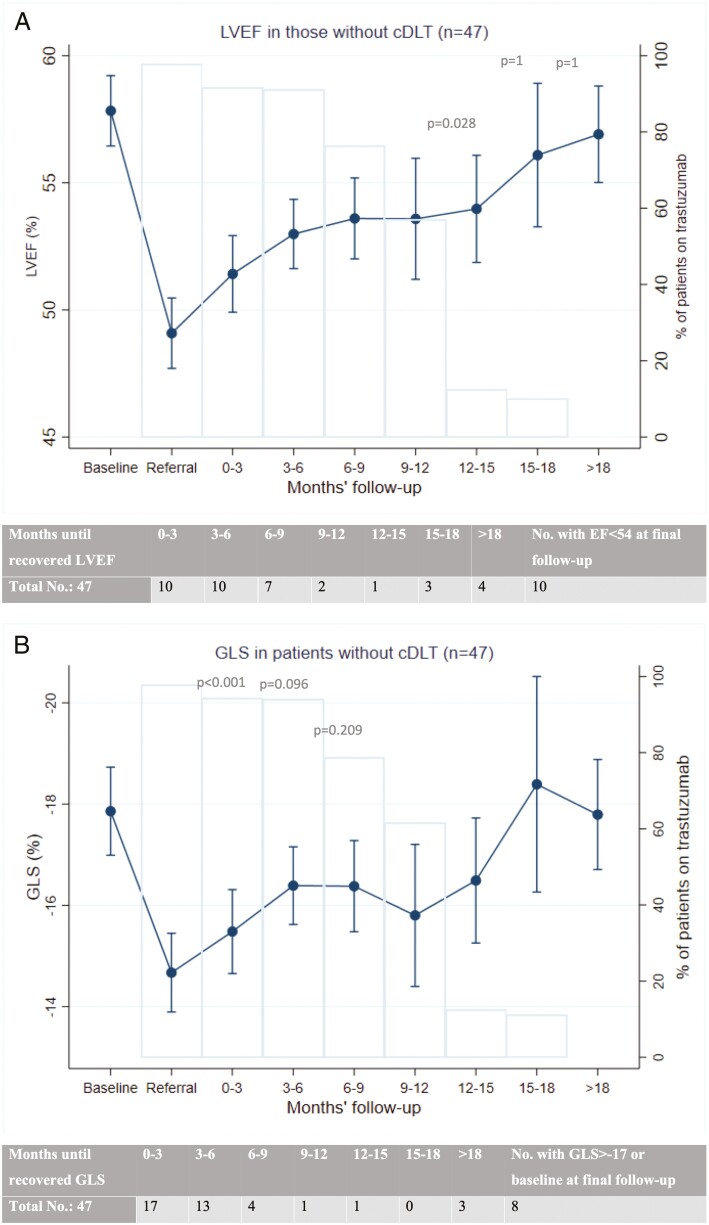
Left ventricular ejection fraction (LVEF) and global longitudinal strain (GLS) in patients without cardiac-dose limiting toxicity (cDLT). The graphs show trend in left ventricular ejection fraction (**a**) and global longitudinal strain (**b**, scale reversed for ease of interpretation) in patients without cDLT (*n* = 47) in the study. Bars represent percent of patients on trastuzumab at each time point. Table accompanying each graph shows the number of patients who demonstrated normalized LVEF (≥54%) or normalized GLS (≤−17% or to baseline level) at each time point. *P*-values represent the comparison of LVEF or GLS with baseline values at selected time points.

**Figure 3.**
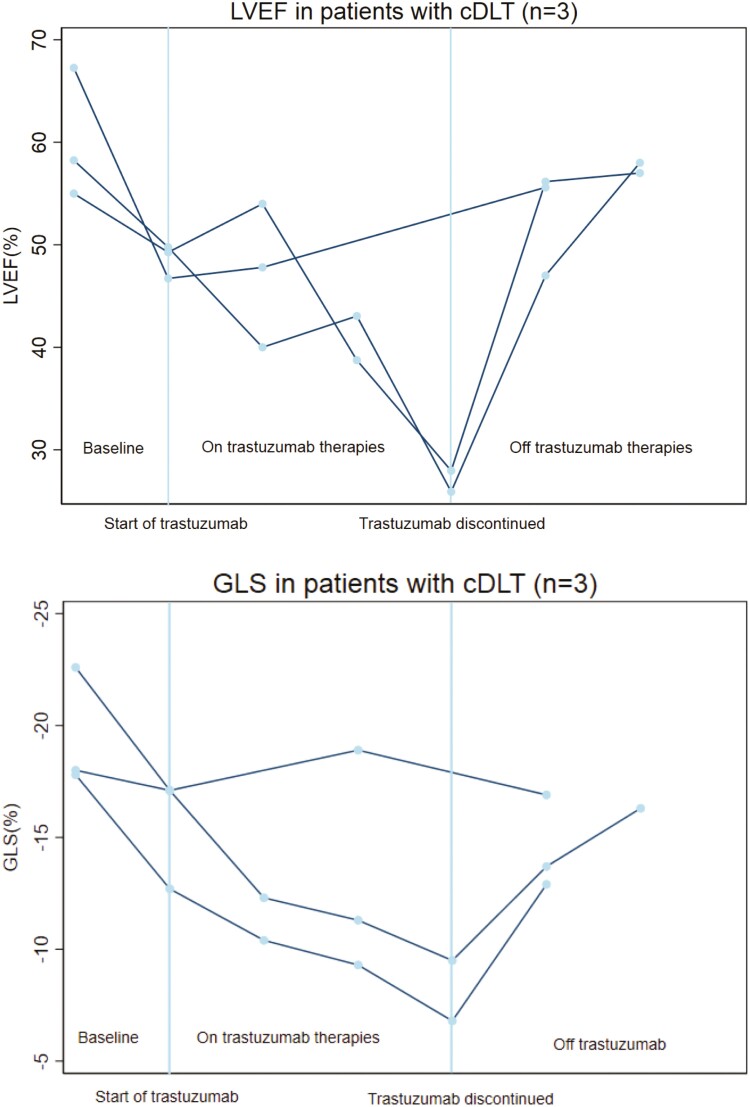
Left ventricular ejection fraction (LVEF) and global longitudinal strain (GLS) in patients with cardiac dose-limiting toxicity (cDLT). Trend in left ventricular ejection fraction and global longitudinal strain (scale reversed for ease of interpretation) from pre-trastuzumab baseline to final follow-up in individuals who developed cDLT (*n* = 3).

LVEF and GLS during follow-up are shown in Figs. 1–3. Of all 51 patients, 78% (40 patients) recovered LVEF to normal range (≥54%) at final follow-up. Of these, 21 (53%) recovered by 6 months and 32 (80%) recovered by 15 months after the initial cardiotoxicity. GLS also recovered to normal range (≤−17%) or to baseline (mean −17.8% ± 2.8) in 78% (40 patients), 45% of those by 3 months and 88% by 9 months after onset of cardiac injury (Fig. 1).

Among those without cDLT (47 patients), LVEF and GLS normalized in 79% (37) and 83% (39) at the final follow-up; the rate of recovery for both is similar to the analyses for all patients above (Fig. 2).

The LVEF and GLS trends for individual patients who experienced cDLT are shown in Fig. 3. At final follow-up after trastuzumab discontinuation, there remained a relative impairment in GLS of 20.7% ± 10 (absolute GLS of −15.4% ± 2.2). The average final LVEF was 56.9% ± 1.2, similar to the LVEF in patients without cDLT (*P* = .67).

The trough LVEF among the individuals experiencing cDLT was 33.0% ± 10.5% as compared with 46.4% ± 4.3% among those who did not develop cDLT (*P* < .001). The maximum relative reduction in GLS was 32.5% ± 8.7% and 23.7% ± 13.6% among the individuals who did and did not develop cDLT respectively (*P* = .23).

### Oncological Outcomes

Five (10%) patients developed new metastatic disease or cancer progression during follow-up and 1 died of breast cancer; all 5 completed planned trastuzumab. In comparison, none of the patients who stopped trastuzumab prematurely due to cDLT developed any disease progression. One patient died of a condition unrelated to breast cancer or cardiovascular disease.

### Sensitivity Analysis

The International Cardio-Oncology Society recently developed a consensus definition of cardiotoxicity, which differs from historical definitions. In total, 45 (88%) patients included in this study met the newly published International Cardio-Oncology consensus (ICOS) definition of cardiotoxicity at the time of referral,^13^ including 11(24%) with mild, 18 (40%) with moderate, and 16 (36%) with severe cardiotoxicity. We performed 2 sets of sensitivity analyses: (1) limited to this subgroup of patients meeting ICOS definition, and (2) limited to patients with non-metastatic disease at the onset of cardiotoxicity. Both showed similar results in baseline characteristic comparisons and clinical outcomes (Supplementary Tables S3–S4, Supplementary Fig. S2–S3).

## Discussion

The major findings from our study are: (1) patients with ­trastuzumab-related cardiac injury undergoing permissive cardiotoxicity may still experience reduced LVEF 3 years after initial treatment for LV dysfunction; (2) 6% of patients developed cDLT during a strategy of permissive cardiotoxicity; (3) patients who developed cDLT during trastuzumab are more likely to have persistent cardiotoxicity despite trastuzumab discontinuation.

### Permissive Cardiotoxicity

Two phase I trials (SCHOLAR and SAFE-HeaRT) demonstrated that continuing trastuzumab with manifest LV systolic dysfunction is feasible. In SaFE-HeART, 30 patients with stages I-IV HER2-positive breast cancer who received trastuzumab with or without pertuzumab or T-DM1 were evaluated. In SCHOLAR, 20 early-stage breast cancer patients on trastuzumab were evaluated. Echocardiograms were performed serially in both trials while participants continued to receive HER2-targeted therapies and cardioprotective medications. Approximately 10% of patients in both trials discontinued trastuzumab due to cDLT. In our current study, 6% of patients experienced cardiac dose-limiting toxicity resulting in premature and permanent trastuzumab cessation. Most were mildly symptomatic but had severe impairment in LVEF (average decline of 15.5%). GLS also declined significantly with an average relative drop of 33%, albeit not used as a criterion for discontinuation. Heart failure hospitalization occurred in 1.9%. and there were no cardiac deaths in our study. In comparison, the HERA trial reported a rate of 0.6% severe heart failure (NYHA III or IV) during 11 years of follow-up.^7^

### Risk Factors for Trastuzumab-Related Cardiotoxicity

Recognized risk factors for developing trastuzumab-related cardiotoxicity include treatment with higher cumulative doses of anthracycline, older age, underlying heart disease, a lower baseline LVEF pre-therapy, radiation to left chest or mediastinum, and a higher body-mass index.^7,18-21^ The Canadian Trastuzumab Working Group and ESC ­Cardio-Oncology guideline recommended special consideration for patients with pre-existing ischemic heart disease or significant valvular heart disease prior to the initiation of treatment with trastuzumab.^7,12^ A retrospective study noted that valvular heart disease may be associated with the risk of developing trastuzumab-related cardiotoxicity.^22^ There is a lack of data available to help predict which patients are at higher risk for developing persistent cardiac dose-limiting toxicity under a strategy of permissive cardiotoxicity with HER2-targeted therapies.

### Intermediate-Term Cardiovascular Outcomes

Long-term follow-up data from the HERA trial up to 12 years post-trastuzumab showed that primary cardiac events (defined as NYHA Class III or IV symptomatic heart failure,with ongoing LV dysfunction or cardiac death) were rare, supporting its long-term safety. NSABP-B31, another large-scale randomized controlled trial, albeit with a different trastuzumab protocol from HERA, similarly showed during 7 years of follow-up that mild cardiotoxicity, defined as NYHA classes I or II with an LVEF drop of >10% to below 50%, occurred in 4.4%.^23,24^ The pooled analysis of NSABP-B31, HERA, and NCCTG 9831 including 7445 patients with over 10 years of median follow-up showed 8.3% developed mildly symptomatic or asymptomatic LVEF decrease, and 2.3% severe congestive heart failure among patients treated with trastuzumab.^25^ However, patients who developed cardiotoxicity while receiving trastuzumab had to withhold or permanently discontinue therapy.^3^ Little is known about the intermediate- and long-term cardiac outcomes for individuals managed with a strategy of permissive cardiotoxicity.

In our study, there was no symptomatic heart failure, ­cardiac-related hospitalization, or death in patients who did successfully undergo permissive cardiotoxicity. However, LVEF did not fully recover 3 years after the initial cardiac injury: 14% (7) of our patients still had ongoing LV dysfunction at final follow-up. Patients who could not tolerate permissive cardiotoxicity were also more likely to have persistent LV dysfunction. As most (92%) of our patients received anthracycline prior to trastuzumab, it is difficult to discern whether the delayed LV recovery was due to anthracycline or trastuzumab toxicity, or both. Yoon et al also noted a 20% rate of persistent cardiotoxicity 6 months after initial LV dysfunction; their patient population similarly had a high rate of anthracycline use.^26^ Decoupling the cardiotoxicity profiles of both therapies is challenging, as their sequential use is common. Regardless of the etiology, the prolonged LV dysfunction seen with permissive cardiotoxicity remains an important observation that warrants further studies.

Interestingly, most of our patients demonstrated an impairment in GLS in addition to LVEF during permissive cardiotoxicity, but GLS appeared to normalize sooner than LVEF after the initial cardiac injury. GLS is thought to be a more sensitive predictor of trastuzumab-related cardiotoxicity, but its measurement is less precise than LVEF which complicates an interpretation of its clinical utility in monitoring LV function.^27^ Studies show that GLS may be a marker of myocardial fibrosis in other types of cardiomyopathy.^28,29^ The rapid GLS recovery in our study may suggest that permissive cardiotoxicity does not lead to permanent myocardial fibrosis and irreversible injury, but full myocardial recovery in the form of LVEF may be more delayed.

### Cardiac Safety of Other HER2-Targeted Therapies

Other HER2-targeted therapies such as pertuzumab and trastuzumab emtansine (T-DM1) are increasingly being used in the management of patients with both early stage and metastatic HER2-positive breast cancer. In a recent meta-analysis that examined 7 breast cancer trials and 1 gastric cancer trial, pertuzumab increased the risk of clinical heart failure, but not asymptomatic left ventricular systolic dysfunction.^30^ The cardiac safety data of T-DM1 from a published cohort of 2000 patients showed a ­cardiac-related adverse event rate of 2.7%; 0.4% of patients dropped LVEF to less than 40%.^31^ The APHINITY trial that studied pertuzumab added to adjuvant trastuzumab and chemotherapy showed a primary cardiac event rate (heart failure and cardiac death) of 0.8% (vs 0.3% in control group) and secondary cardiac event rate (mildly symptomatic or asymptomatic LVEF decrease) similar to control group at 2.7%.^3^ In our study, all patients who were on combined trastuzumab and pertuzumab, or received T-DM1 after trastuzumab were able to complete both therapies despite manifest reduction in LV systolic function. In patients with metastatic breast cancer who may benefit from combined HER2-targeted therapies, a strategy of permissive cardiotoxicity can also be applied.

### Study Strengths and Limitations

To our knowledge, this study represents the largest and longest report of outcomes after implementation of permissive cardiotoxicity. However, in absolute terms, our sample size is limited, which precludes a more robust statistical analysis and power to identify other differences between those who did versus did not develop cDLT. The study design was retrospective and from a single center so therefore may not be widely generalizable. Finally, the definition of cardiotoxicity according to various published guidelines has changed as the field of Cardio-Oncology evolves. What was considered cardiotoxicity previously may no longer hold.

## Supplementary Material

Supplementary material is available at *The Oncologist* online.

oyad086_suppl_Supplementary_Material

## Data Availability

The data underlying this article will be shared on reasonable request to the corresponding author.

## Abbreviations:

ACEI: angiotensin converting enzyme inhibitor
ARB: angiotensin receptor blocker
cDLT: cardiac dose-limiting toxicity
GLS: global longitudinal strain
HER2: human epidermal growth factor 2
LVEF: left ventricular ejection fraction
NYHA: New York Heart Association
T-DM1: Trastuzumab emtansine
