# Supplementary material for: Three-Year Outcomes Following Permissive Cardiotoxicity in Patients on Trastuzumab
Source: Oncologist. 2023 Apr 24;28(9):e712–22. doi: 10.1093/oncolo/oyad086 (PMC10485282; doi:10.1093/oncolo/oyad086)

Three-Year Outcomes Following Permissive Cardiotoxicity in Patients on Trastuzumab:

Supplementary materials

Supplementary Figure 1

Supplementary Table 1:

Baseline characteristics of patients with or without evidence of cardiotoxicity as defined by ICOS criteria at final follow-up. The patient who discontinued trastuzumab by preference was not included in the comparative analysis.

|  | Without cardiotoxicity  N=43 (86%) | With  cardiotoxicity  N= 7 (14%) | p-value |
| --- | --- | --- | --- |
| Age | 54.9 ± 10.5 | 62.0 ± 9.6 | 0.10 |
| Left-sided radiation | 18 (36) | 3 (2) | 0.96 |
| Received anthracycline-based therapies | 39 (78) | 7 (14) | 0.41 |
| Cumulative doxorubicin-equivalent dose, mg/m^2^ | 238.0±22.5 | 276.7 ± 105.0 | 0.04 |
| BMI, kg/m^2^ | 28.6±7.1 | 31.8±8.0 | 0.27 |
| Systolic BP, mmHg | 123.9± 13.0 | 120.6±8.9 | 0.55 |
| Diastolic BP, mmHg | 75.5±4.3 | 75.5±7.1 | 0.99 |
| Coronary Artery Disease | 4 (8) | 0 (0) | 0.41 |
| Alcohol >10 drinks/week | 2 (4) | 1 (2) | 0.33 |
| Current smoking | 2 (4) | 1 (2) | 0.33 |
| Dyslipidemia | 6 (12) | 3 (6) | 0.07 |
| Hypertension | 10 (20) | 3 (6) | 0.28 |
| Diabetes Mellitus | 3 (6) | 1(2) | 0.52 |
| Any cardiovascular risk factor* | 19 (38) | 4(8) | 0.53 |
| Time from trastuzumab initiation to onset of cardiotoxicity (days) | 152.5±135.4 | 174.8±130.3 | 0.71 |
| LVEF pre-HER2 therapy, % | 57.6±4.8 | 60.1±3.3 | 0.19 |
| LVEF at cardio-oncology referral | 48.3±4.2 | 47.8±4.6 | 0.77 |
| LVESV index pre-HER2 therapy (mL/m^2^) | 20.4±5.1 | 18.6±7.0 | 0.45 |
| TAPSE, cm | 2.0±0.3 | 2.1±0.6 | 0.77 |
| ≥ moderate valve disease pre-HER2 therapy | 0 | 1 (2) | 0.01 |

* includes smoking, dyslipidemia, diabetes, hypertension, coronary artery disease

Table 3. LV function in patients who had persistent cardiotoxicity after trastuzumab completion or discontinuation

| ID | Baseline GLS | Nadir GLS | GLS at final follow-up | Baseline LVEF | Nadir LVEF | LVEF at final follow-up | Worst NYHA | Follow-up (days) | Pre-existing cardio-myopathy | Discontinued trastuzumab prematurely due to cDLT |
| --- | --- | --- | --- | --- | --- | --- | --- | --- | --- | --- |
| 16 | -21.8 | -15 | -16.9 | 58 | 50 | 51.29 | I | 155 | 0 | No |
| 23 | -17.8 | -6.8 | -12.9 | 58.24 | 25.91 | 58 | III | 1416 | 0 | Yes |
| 28 | -20.2 | -14.1 | -14.9 | 58.02 | 50 | 53.14 | I | 602 | 0 | No |
| 37 | N/A | -12.4 | -16.6 | 58.47 | 45 | 47 | II | 759 | 0 | No |
| 38 | -21.8 | -11.5 | -16.7 | 61 | 38.07 | 52.78 | I | 974 | 0 | No |
| 39 | N/A | -14.1 | -14.2 | 60 | 45.4 | 48.69 | I | 155 | 0 | No |
| 43 | -22.6 | -16.9 | -16.9 | 67.26 | 45 | 55.6 | II | 256 | 0 | Yes |

Supplementary Table 3: Baseline characteristics of patients meeting ICOS definition of cardiotoxicity, stratified by the occurrence of cardiac dose-limiting toxicity (cDLT). The patient who elected not complete HER2-targeted therapy (but did not have cDLT) is not included in this analysis.

|  | Overall  N= 44 (100%) | With cDLT  N=3 (7%) | Without cDLT  N= 41 (93%) | p-value |
| --- | --- | --- | --- | --- |
| Age | 56.9 ± 10.8 | 57.3 ± 15.5 | 56.9 ± 10.4 | 0.95 |
| Left-sided radiation | 21 (46.7) | 2 (50) | 19 (46.3) | 0.89 |
| Received anthracycline-based therapies | 41 (91.1) | 1 (100) | 37 (90.2) | 0.52 |
| Cumulative doxorubicin-equivalent dose, mg/m^2^ | 247.1±56.7 | 342± 167.3 | 236.9± 19.8 | 0.001 |
| BMI, kg/m^2^ | 29.4±7.2 | 26.5±0.18 | 29.8±7.5 | 0.4 |
| Systolic BP, mmHg | 123.6± 13.1 | 122.4± 11.3 | 123.7± 13.4 | 0.85 |
| Diastolic BP, mmHg | 75.4± 7.0 | 74.8±8.7 | 75.5±7 | 0.86 |
| Coronary Artery Disease | 5 (10.9) | 1 (25) | 4 (9.8) | 0.37 |
| Alcohol >10 drinks/week | 3 (6.7) | 1 (25) | 2 (4.9) | 0.13 |
| Current smoking | 2 (4.4) | 0 | 2 (4.9) | 0.66 |
| Dyslipidemia | 9 (20) | 1 (25) | 8 (20) | 0.8 |
| Hypertension | 12 (26.7) | 2 (50) | 10 (24.4) | 0.28 |
| Diabetes | 4 (8.9) | 1 (25) | 3 (7.3) | 0.25 |
| Any heart failure risk factor* | 23(51.1) | 3(75) | 20(48.8) | 0.33 |
| Time to onset of cardiotoxicity (days) | 136.8±80.6 | 123.3±56 | 138.3±83.4 | 0.73 |
| GLS pre-HER2 therapy, % | -18.3±2.9 | -19.5±2.7 | -18.2±2.9 | 0.48 |
| LVEF pre-HER2 therapy, % | 58.6±4.9 | 61.6±6 | 58.3±4.7 | 0.19 |
| LVEF at cardio-oncology referral | 48±4.3 | 45.3±4.1 | 48.2±4.3 | 0.19 |
| LVESV pre-HER2 therapy | 19.74±5.5 | 20.2±6.9 | 19.7±5.5 | 0.89 |
| TAPSE, cm | 2.1±0.35 | 2±0.32 | 2.1±0.36 | 0.5 |
| ≥ moderate valve disease pre-HER2 therapy | 1 (2.2) | 1(25) | 0 | <0.001 |

* includes smoking, dyslipidemia, diabetes, hypertension, coronary artery disease

Table 4a: Baseline characteristics of patients with or without cardiac dose-limiting toxicity, with non-metastatic disease only. The patient who discontinued trastuzumab by preference was not included in the comparative analysis.

|  | Without cardiotoxicity  N 40= (%) | With  cardiotoxicity  N= 3 (%) | p-value |
| --- | --- | --- | --- |
| Age | 56±10.6 | 57±17.9 | 0.84 |
| Left-sided radiation | 17 (40) | 1 (2) | 0.76 |
| Received anthracycline-based therapies | 39 (91) | 3 (7) | 0.79 |
| Cumulative doxorubicin-equivalent dose, mg/m^2^ | 232.6 ±17.1 | 343.1±144.8 | <0.01 |
| BMI, kg/m^2^ | 30.2±7.4 | 26.5±0.2 | 0.40 |
| Systolic BP, mmHg | 123.2±12.9 | 126.4±9.4 | 0.67 |
| Diastolic BP, mmHg | 74.9±6.8 | 79.1±2.0 | 0.29 |
| Coronary Artery Disease | 3 (7) | 0 | 0.63 |
| Alcohol >10 drinks/week | 2 (5) | 1 (2) | 0.07 |
| Current smoking | 3 (7) | 0 | 0.63 |
| Dyslipidemia | 7 (16) | 1 (2) | 0.51 |
| Hypertension | 9 (21) | 2 (5) | 0.10 |
| Diabetes Mellitus | 3 (7) | 1 (2) | 0.14 |
| Any cardiovascular risk factor* | 19 (44) | 2 (5) | 0.53 |
| Time from trastuzumab initiation to onset of cardiotoxicity (days) | 139.5 ± 81.5 | 140 ±54.9 | 0.99 |
| LVEF pre-HER2 therapy, % | 58.0±4.6 | 60.2±6.4 | 0.44 |
| LVEF at cardio-oncology referral | 48.4±3.6 | 44.6±4.8 | 0.10 |
| LVESV index pre-HER2 therapy (mL/m^2^) | 20.1±5.4 | 20.2±6.9 | 0.98 |
| TAPSE, cm | 2.1±0.36 | 2.0±0.32 | 0.58 |
| ≥ moderate valve disease pre-HER2 therapy | 0 | 1(2) | <0.01 |

Table 4b: Baseline characteristics of patients with non-metastatic disease, with or without cardiotoxicity (by ICOS definition) at final follow-up. The patient who discontinued trastuzumab by preference was not included in the comparative analysis.

|  | Without cardiotoxicity  N= 36(84%) | With  cardiotoxicity  N= 7 (16%) | p-value |
| --- | --- | --- | --- |
| Age | 54.7±10.9 | 62±9.6 | 0.11 |
| Left-sided radiation | 15 (35) | 3 (7) | 0.95 |
| Received anthracycline-based therapies | 35 (81) | 7 (16) | 0.66 |
| Cumulative doxorubicin-equivalent dose, mg/m^2^ | 233.3 ±17.1 | 276.7±105.0 | 0.02 |
| BMI, kg/m^2^ | 29.6±7.1 | 31.8±8.0 | 0.5 |
| Systolic BP, mmHg | 123.9±13.2 | 120.6±8.9 | 0.6 |
| Diastolic BP, mmHg | 75.1±7.0 | 75.5±4.3 | 0.91 |
| Coronary Artery Disease | 3 (7) | 0 | 0.44 |
| Alcohol >10 drinks/week | 2 (5) | 1 (2) | 0.42 |
| Current smoking | 2 (5) | 1 (2) | 0.42 |
| Dyslipidemia | 5 (12) | 3 (7) | 0.07 |
| Hypertension | 8 (19) | 3 (7) | 0.26 |
| Diabetes Mellitus | 3 (7) | 1 (2) | 0.63 |
| Any cardiovascular risk factor* | 17 (40) | 4 (10) | 0.64 |
| Time from trastuzumab initiation to onset of cardiotoxicity (days) | 133.2±67.3 | 174.8±113.3 | 0.24 |
| LVEF pre-HER2 therapy, % | 57.7±4.9 | 60.1±3.3 | 0.22 |
| LVEF at cardio-oncology referral | 48.2±3.6 | 47.8±4.6 | 0.76 |
| LVESV index pre-HER2 therapy (mL/m^2^) | 20.4±5.2 | 18.6±5.5 | 0.45 |
| TAPSE, cm | 2.1±0.3 | 2.1±0.6 | 0.82 |
| ≥ moderate valve disease pre-HER2 therapy | 0 | 1 (2) | 0.02 |

Supplementary Figure 2a: left ventricular ejection fraction in patients who met ICOS criteria of cardiotoxicity and completed permissive cardiotoxicity without dose-limiting toxicity (cDLT). Bars represent percent of patients on trastuzumab at each time point.


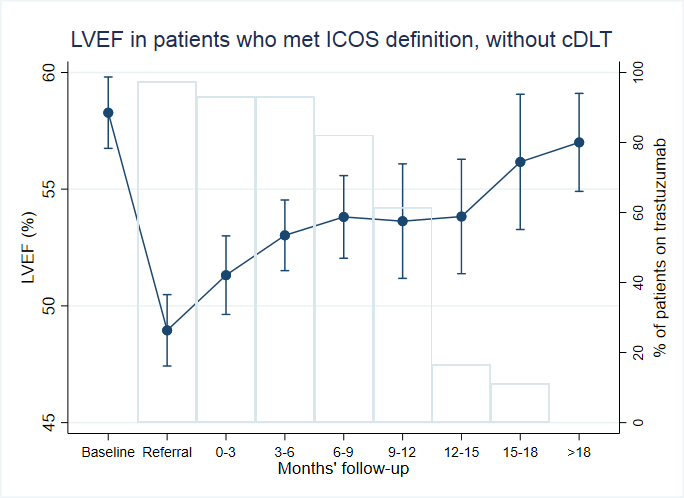


Supplementary Figure 2b: Global longitudinal strain (scale reversed for ease of interpretation) in patients who met ICOS criteria of cardiotoxicity and completed permissive cardiotoxicity without dose-limiting toxicity (cDLT). Bars represent percent of patients on trastuzumab at each time point.


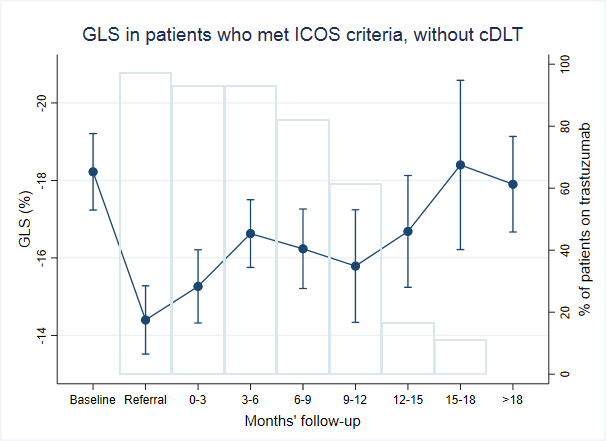


Supplementary Figure 3a

left ventricular ejection fraction in patients with non-metastatic disease at the time of initial cardiotoxicity (n=44). Bars represent percent of patients on trastuzumab at each time point.


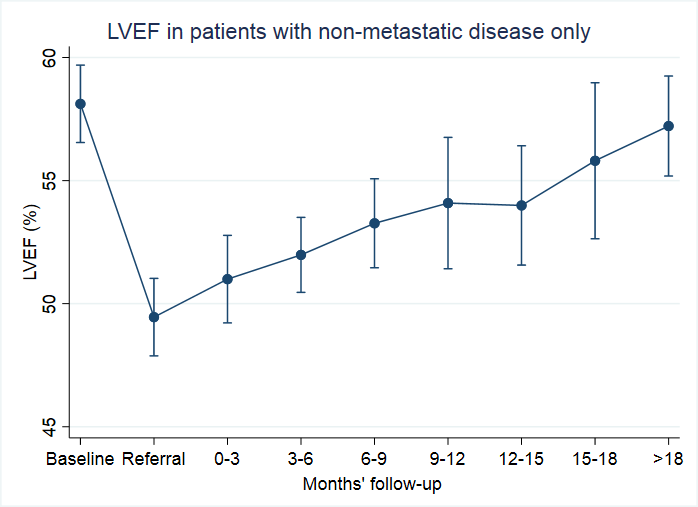


Supplementary Figure 3b

Global longitudinal strain (scale reversed for ease of interpretation) in patients with non-metastatic disease at the time of initial cardiotoxicity (n=44). Bars represent percent of patients on trastuzumab at each time point.


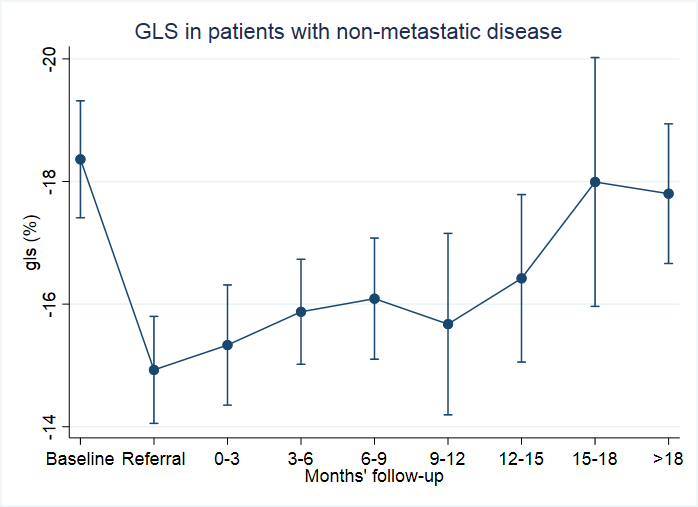

Supplement: oyad086_suppl_Supplementary_Material [file oyad086_suppl_supplementary_material.docx]
